# Indoleamine 2,3-dioxygenase 1 in cancer immunotherapy: from small-molecule inhibition to PROTAC-mediated degradation

**DOI:** 10.3389/fphar.2025.1640073

**Published:** 2025-08-12

**Authors:** Xiuyun Li, Hao Meng, Hefeng Wang, Yujing Zhang, Wanpeng Yu

**Affiliations:** ^1^ Infection and Microbiology Research Laboratory for Women and Children, Shandong Provincial Maternal and Child Health Care Hospital Affiliated to Qingdao University, Jinan, Shandong, China; ^2^ The Affiliated Cardiovascular Hospital of Qingdao University, Qingdao University, Qingdao, Shandong, China; ^3^ Qingdao Medical College, Qingdao University, Qingdao, China

**Keywords:** IDO1, small-molecule inhibitors, PROTACs, protein degradation, cancer immunotherapy

## Abstract

Indoleamine 2,3-dioxygenase 1 (IDO1) has emerged as a critical immunometabolic regulator in cancer, orchestrating immunosuppression through its rate-limiting catabolism of tryptophan to kynurenine. This enzymatic activity establishes an immunosuppressive tumor microenvironment via two distinct pathways: GCN2-mediated T cell anergy resulting from tryptophan depletion, and AhR-dependent immune tolerance induced by accumulating kynurenine metabolites. The therapeutic landscape of IDO1 inhibition has progressed significantly from early heme-competitive inhibitors like epacadostat to next-generation proteolysis-targeting chimera (PROTAC) technology. While over 20 small-molecule IDO1 inhibitors have entered clinical trials for various cancers, their variable efficacy has underscored the need for improved target engagement strategies and better patient selection biomarkers. PROTACs represent a paradigm shift in IDO1 modulation, offering the unique advantage of complete target degradation rather than mere inhibition. This review systematically evaluates: (1) clinically investigated IDO1 inhibitors and their pharmacological profiles, and (2) the preclinical promise of IDO1-targeting PROTAC degraders. Through critical analysis of their mechanisms of action and therapeutic potential, we provide insights into optimizing IDO1-targeted strategies for cancer immunotherapy.

## 1 Introduction

The field of cancer immunotherapy has witnessed transformative progress in the past decade, particularly with the clinical success of immune checkpoint inhibitors and adoptive cell therapies (Dall'Olio et al., 2022; Arafat Hossain, 2024; Dhasmana et al., 2023). Despite these advancements, the immunosuppressive tumor microenvironment (TME) remains a formidable barrier to achieving sustained therapeutic responses across diverse malignancies. The TME represents a complex ecosystem where cancer cells employ sophisticated mechanisms to evade immune surveillance, with metabolic reprogramming playing a particularly crucial role in immune suppression (Morad et al., 2021; Bagchi et al., 2021). Among the various immunoregulatory pathways, the indoleamine 2,3-dioxygenase 1 (IDO1)-mediated kynurenine pathway has emerged as a central player in tumor immune evasion. This heme-containing enzyme catalyzes the rate-limiting step in tryptophan catabolism, converting this essential amino acid into N-formylkynurenine and subsequently to kynurenine metabolites. The immunomodulatory effects of IDO1 activity operate through two distinct but complementary mechanisms: firstly, tryptophan depletion triggers the amino acid starvation response via GCN2 kinase activation, leading to T cell dysfunction and apoptosis; secondly, the accumulating kynurenine metabolites activate the aryl hydrocarbon receptor (AhR), driving the differentiation of immunosuppressive regulatory T cells (Tregs) while simultaneously impairing effector T cell function (Fujiwara et al., 2022; Nawas et al., 2023).

Given its pivotal role in establishing an immunosuppressive TME, IDO1 has become a focus of intense investigation as a therapeutic target in oncology (Tang et al., 2021; Zhai et al., 2018). The initial excitement surrounding IDO1 inhibition was supported by robust preclinical data demonstrating that pharmacological intervention could effectively reverse tumor-induced immune tolerance and potentiate the effects of other immunotherapeutic modalities. However, the translation of these promising preclinical findings into clinical success has been unexpectedly challenging (Cheong and Sun, 2018; Dey et al., 2021). Several high-profile clinical trials evaluating small-molecule IDO1 inhibitors have reported inconsistent outcomes, with many failing to meet their primary endpoints. These clinical setbacks have prompted a thorough reevaluation of IDO1 targeting approaches and underscored the need for more sophisticated therapeutic strategies (Panfili et al., 2023; Rossini et al., 2024). In this evolving landscape, proteolysis-targeting chimera (PROTAC) technology has emerged as a groundbreaking alternative to conventional inhibition. PROTACs represent a paradigm shift in drug development by harnessing the cell’s natural protein degradation machinery to achieve complete and sustained target elimination (Li et al., 2023; Wang et al., 2021). Unlike traditional inhibitors that merely block enzymatic activity, PROTAC-mediated degradation offers several theoretical advantages: it not only abolishes IDO1’s catalytic function but also eliminates potential non-enzymatic roles of the protein, may prevent compensatory feedback mechanisms, and could provide more durable pharmacological effects. Recent preclinical studies with IDO1-directed PROTACs have shown promising results in remodeling the immunosuppressive TME, potentially addressing the limitations observed with standard inhibitors (Tang et al., 2022; Wang et al., 2021). This review provides a comprehensive analysis of IDO1-targeted therapeutic development, examining both the clinical experience with conventional inhibitors and the emerging potential of PROTAC-based degraders, while discussing their implications for the future of cancer immunotherapy.

## 2 IDO1 as a key immunometabolic checkpoint in cancer

### 2.1 IDO1-mediated immunosuppression in the tumor microenvironment

Indoleamine 2,3-dioxygenase 1 (IDO1) has emerged as a pivotal regulator of tumor immune evasion through its intricate modulation of tryptophan (Trp) catabolism. This heme-containing enzyme initiates the rate-limiting step in the kynurenine pathway, converting Trp to N-formylkynurenine, which is subsequently metabolized to kynurenine (Kyn) and downstream metabolites (Tang et al., 2021). The immunosuppressive effects of IDO1 are mediated through two interconnected mechanisms: (1) Trp depletion-induced activation of the general control nonderepressible 2 (GCN2) kinase pathway in effector T cells, leading to phosphorylation of eukaryotic initiation factor 2α (eIF2α) and subsequent cell cycle arrest; and (2) Kyn-mediated activation of the aryl hydrocarbon receptor (AhR), which promotes Foxp3+ regulatory T cell differentiation while inhibiting Th17 development (Xiang et al., 2020). These molecular events collectively establish an immunosuppressive niche that facilitates tumor immune escape and confers resistance to immune checkpoint inhibitors.

### 2.2 Clinical correlates and regulation of IDO1 in human malignancies

Comprehensive immunohistochemical studies have demonstrated significant IDO1 overexpression across diverse malignancies, with particularly high expression observed in triple-negative breast cancer (TNBC), glioblastoma multiforme (GBM), and microsatellite-stable colorectal carcinomas (CRC) (Muller et al., 2019). The enzyme’s expression pattern exhibits remarkable spatial heterogeneity, with predominant localization in tumor-infiltrating immune cells and tumor-associated stromal components rather than malignant cells themselves. This compartmentalization suggests a paracrine mechanism of immune suppression. Importantly, IDO1 expression is dynamically regulated by inflammatory mediators, particularly interferon-γ (IFN-γ) through JAK1/2-STAT1/3 signaling, creating a feed-forward loop wherein immune cell infiltration inadvertently enhances local immunosuppression (Ge et al., 2020). Clinical outcome analyses reveal that elevated tumoral IDO1 expression or increased serum Kyn/Trp ratio correlates with reduced progression-free survival across multiple cancer types, independent of other prognostic factors.

### 2.3 Therapeutic targeting and translational implications

The central role of IDO1 in tumor immune evasion has spurred development of multiple pharmacological inhibitors, including epacadostat (INCB024360) and indoximod (1-methyl-D-tryptophan), which have shown promising preclinical efficacy in combination with PD-1/PD-L1 blockade (Zeitler and Murray, 2023). However, recent clinical trials have highlighted the complexity of targeting this pathway, with the ECHO-301 trial demonstrating no survival benefit from epacadostat-pembrolizumab combination in melanoma. These findings underscore the need for better patient stratification strategies, potentially incorporating IDO1 pathway activity biomarkers such as tumoral IDO1 mRNA expression or circulating Kyn levels. Emerging evidence also suggests that IDO1 inhibition may synergize with conventional therapies, particularly in tumors with high baseline inflammatory signatures (Sarangi, 2023). Further investigation is warranted to elucidate optimal combinatorial approaches and identify predictive biomarkers for IDO1-targeted therapies.

## 3 Clinical development of IDO1 inhibitors

The strategic inhibition of tryptophan metabolism through interference with IDO1 and TDO enzymatic activity has emerged as a significant focus in cancer immunotherapy research. Multiple pharmacological agents targeting these pathways have progressed through various stages of clinical evaluation, as comprehensively documented in Table 1 (Van den Eynde et al., 2020; Platten et al., 2019). Among these, epacadostat represents a prototypical IDO1 inhibitor that functions through competitive displacement of tryptophan at the enzyme’s active site. Its clinical development was initiated following compelling preclinical evidence demonstrating enhanced T-cell and natural killer cell functionality and proliferation (Liu et al., 2010; Sieviläinen et al., 2022). Initial human studies confirmed the agent’s favorable safety profile in patients with advanced malignancies, though its antitumor efficacy as monotherapy proved limited (Beatty et al., 2013). Building on observations of synergistic activity with immune checkpoint modulators in experimental systems (Wainwright et al., 2014), subsequent clinical investigations explored combination regimens incorporating anti-CTLA-4 and anti-PD-L1 antibodies, yielding encouraging preliminary outcomes (Beatty et al., 2017). The phase 1/2 ECHO-202/KEYNOTE-037 trial, employing an open-label, single-arm design with dose escalation, evaluated epacadostat in combination with pembrolizumab across various advanced solid tumors. This study reported notable objective response rates (40.3% overall, with particularly impressive 61.9% responses in melanoma patients) (Mitchell et al., 2018). However, these promising signals were not substantiated in subsequent phase 3 confirmatory trials (Long et al., 2019), highlighting the challenges in translating early-phase results to definitive clinical benefit.

**TABLE 1:** Current status of development of representative IDO1 inhibitors in clinical trials (PubMed and Clinicaltrials.gov).

Drugs	Phase	Target	Cancer types	NCT number
Epacadostat (INCB024360)	Phase I/II	IDO1/TLR3	Fallopian Tube Carcinoma Ovarian Carcinoma Primary Peritoneal Carcinoma	NCT02166905
Epacadostat (INCB024360) + Pembrolizumab	Phase II	IDO1/PD-1	Sarcoma	NCT03414229
Epacadostat (INCB024360) + Pembrolizumab	Phase I/II	IDO1/CTLA-4	Melanoma	NCT01604889
Epacadostat (INCB024360) + Cyclophosphamide	Phase I/II	IDO1	Epithelial Ovarian Cancer Fallopian Tube Cancer Recurrent Peritoneal Cancer	NCT02785250
Epacadostat (INCB024360) + SHR9146 + SHR-1210	Phase I	IDO1	Solid Tumors Metastatic Neoplasm Malignant	NCT03491631
Epacadostat (INCB024360) + Itacitinib	Phase I	IDO1/JAK1	Solid Tumors	NCT02559492
Epacadostat (INCB024360) + Nivolumab	Phase I	IDO1/PD-1	Advanced Cancer	NCT03335540
Epacadostat (INCB024360) + Pemigatinib	Phase II	IDO1/FGFR	Endometrial Cancer	NCT04463771
Indoximod	Phase II	IDO1	Metastatic Prostate Cancer	NCT01560923
Indoximod + Idarubicin + Cytarabine	Phase I	IDO1	Acute Myeloid Leukemia	NCT02835729
Indoximod + Nab-Paclitaxel + Gemcitabine	Phase I/II	IDO1	Metastatic Pancreatic Adenocarcinoma Metastatic Pancreatic Cancer	NCT02077881
Indoximod + Temozolomide	Phase I	IDO1	Glioblastoma Multiforme Glioma Gliosarcoma	NCT02502708
Indoximod + Pembrolizumab + Nivolumab	Phase II	IDO1	Melanoma	NCT03301636
Indoximod +1-methyl-D-tryptophan	Phase I	IDO1	Breast Cancer Lung Cancer Melanoma Pancreatic Cancer	NCT00739609
Indoximod + Ipilimumab + Nivolumab + Pembrolizumab	Phase I/II	IDO1/PD-1/CTLA-4	Metastatic Melanoma Stage III Melanoma Stage IV Melanoma	NCT02073123
Indoximod + Ibrutinib + Cyclophosphamide + Etoposide	Phase I	IDO1	Metastatic Melanoma Stage III Melanoma Stage IV Melanoma	NCT05106296
Indoximod + Docetaxel + Placebo + Paclitaxel	Phase II	IDO1	Metastatic Breast Cancer	NCT01792050
Indoximod + Radiation	Phase II	IDO1	Glioblastoma Medulloblastoma Ependymoma	NCT04049669
Indoximod + Temozolomide + Bevacizumab	Phase I/II	IDO1/VEGF	Glioblastoma Multiforme Gliosarcoma Malignant Brain Tumor	NCT02052648
NLG802	Phase I	IDO1	Solid Tumors	NCT03164603
Linrodostat (BMS-986205)	Phase II	IDO1	Cancer	NCT03247283
Linrodostat (BMS-986205) + Relatlimab + Nivolumab	Phase I	IDO1/LAG-3/CTLA-4	Advanced Cancer	NCT03459222
Linrodostat (BMS-986205) + Nivolumab	Phase I/II	IDO1/PD-1	Endometrial Adenocarcinoma Endometrial Carcinosarcoma	NCT04106414
Linrodostat (BMS-986205) + Nivolumab	Phase I/II	IDO1/PD-1	Advanced Cancer	NCT03792750
Linrodostat (BMS-986205) + Nivolumab + Placebo	Phase III	IDO1/PD-1	Melanoma Skin Cancer	NCT03329846
Linrodostat (BMS-986205) + Nivolumab + Radiation	Phase I	IDO1/PD-1	Glioblastoma	NCT04047706
Linrodostat (BMS-986205) + Nivolumab + Ipilimumab	Phase II	IDO1/PD-1/CTLA-4	Melanoma Stage III Melanoma Stage IV	NCT04007588
Linrodostat (BMS-986205) + Itraconazole + Rifampin	Phase I	IDO1	Malignancies Multiple	NCT03346837
Linrodostat (BMS-986205) + Nivolumab + Relatlimab + Cabiralizumab + Ipilimumab	Phase I	IDO1/PD-1/LAG-3/CTLA-4	Advanced Cancer	NCT03335540
Linrodostat (BMS-986205) + Nivolumab + Relatlimab + BMS-813160 + Ipilimumab	Phase II	IDO1/PD-1/LAG-3/CTLA-4	Advanced Cancer	NCT02996110
Linrodostat (BMS-986205) + Nivolumab + Cetuximab + Cisplatin + Carboplatin + Fluorouracil	Phase III	IDO1/PD-1/IgG1	Head and Neck Cancer	NCT03386838
Linrodostat (BMS-986205) + Nivolumab + Relatlimab + Rucaparib + Ipilimumab	Phase II	IDO1/PD-1/LAG-3/CTLA-4	Advanced Gastric Cancer	NCT02935634
Linrodostat (BMS-986205) + Nivolumab + Relatlimab + Dasatinib + Ipilimumab	Phase II	IDO1/PD-1/LAG-3/CTLA-4	Advanced Cancer	NCT02750514
Navoximod	Phase I	IDO1	Recurrent Advanced Solid Tumors	NCT20248709
Navoximod + NLG802 + Radiation	Phase I	IDO1	Advanced Solid Tumors	NCT05469490
DN1406131 + Placebo	Phase I	IDO1/TDO	Advanced Solid Tumors	NCT03641794
HTI-1090 (SHR-9146)	Phase I	IDO1/TDO	Advanced Solid Tumors	NCT03208959
IO102-IO103	Phase III	IDO1/PD-1	Metastatic Melanoma Unresectable Melanoma	NCT05155254
KHK-2455 + Avelumab	Phase I	IDO1/PD-1	Urothelial carcinoma	NCT03915405
KHK-2455 + Mogamulizumab	Phase I	IDO1/CCR4	Solid Tumors	NCT02867007
PF-06840003	Phase I	IDO1	Oligodendroglioma Astrocytoma Malignant Glioma	NCT02764151
LPM-3480226	Phase I	IDO1	Solid Tumors	NCT03844438
LY3381916 + LY3300054	Phase I	IDO1	Solid Tumors Non-Small Cell Lung Cancer Renal Cell Carcinoma Triple Negative Breast Cancer	NCT03343613
INCB024360 + Nivolumab + MK-4166 + Ipilimumab	Phase I	IDO1/PD-1 GITR/CTLA-4	Glioblastoma Glioblastoma Multiforme	NCT03707457

Beyond conventional enzymatic inhibitors, alternative approaches to modulating the IDO pathway have been explored. Indoximod and its prodrug formulation NLG802 represent a distinct therapeutic class whose precise mechanism of action continues to be elucidated (Kumar et al., 2020). Early clinical evaluation demonstrated acceptable tolerability when combined with docetaxel in advanced solid tumors (Soliman et al., 2014), though a randomized phase 2 investigation in breast cancer patients failed to demonstrate improved progression-free survival compared to taxane-based therapy alone (Mariotti et al., 2021). Another notable compound, BMS-986205 (linrodostat), distinguished itself as the first IDO1 inhibitor to achieve substantial reductions in circulating kynurenine levels in clinical testing. Subsequent trials combining this agent with nivolumab showed encouraging response rates (34%) in advanced bladder cancer cohorts (Siu et al., 2017; Tabernero et al., 2018), though as with other agents in this class, results across different tumor types have been inconsistent. The collective clinical experience with these various IDO pathway modulators, thoroughly summarized in Table 1, has revealed significant challenges in achieving reproducible therapeutic benefits, particularly in randomized controlled settings.

The initially promising data from early-phase combination trials, particularly the ECHO-202/KEYNOTE-037 study (Mitchell et al., 2018), prompted initiation of the large-scale phase 3 ECHO-301/KEYNOTE-252 trial. This investigation directly compared the epacadostat-pembrolizumab combination (using 100 mg twice daily and 200 mg every 3 weeks dosing, respectively) against pembrolizumab monotherapy in advanced melanoma. The trial outcomes revealed no significant improvements in either progression-free or overall survival with the combination approach (Long et al., 2019). Comprehensive subgroup analyses failed to identify any correlation between PD-L1 expression levels (assessed by immunohistochemistry) and treatment outcomes. Notably, when employing a 1% threshold for positivity, approximately 90% of tumors demonstrated IDO1 expression, yet this marker showed no predictive value for clinical benefit. The study’s inability to establish meaningful pharmacokinetic-pharmacodynamic relationships or identify reliable biomarkers significantly limited insights into the underlying reasons for the combination’s lack of efficacy in this patient population (Long et al., 2019).

Following the ECHO-301/KEYNOTE-252 results, multiple phase 3 trials investigating epacadostat-pembrolizumab combinations across various malignancies were either terminated or scaled back to phase 2 investigations. While certain patient subsets, particularly those with cisplatin-ineligible urothelial carcinoma (31.8% vs. 24.5% ORR) or recurrent advanced urothelial carcinoma (21.4% vs. 9.5% ORR), showed numerically improved response rates with the combination approach, these differences failed to translate into clinically meaningful benefits (Mariotti et al., 2021; Kristeleit et al., 2017). Similar challenges emerged with other IDO1 inhibitors, leading to discontinuation of phase 3 trials evaluating BMS-986205-nivolumab combinations in melanoma, head and neck cancer, and non-small cell lung cancer (NCT03329846, NCT03386838, and NCT03417037), though investigation continues in muscle-invasive bladder cancer (NCT03329846).

Despite the mixed clinical outcomes observed with IDO1 inhibitors in late-phase trials, ongoing research continues to explore their therapeutic potential through refined strategies, including biomarker-driven patient selection, novel combination approaches, and optimized treatment sequencing. Current investigations are evaluating IDO1 inhibition in specific tumor types with high immunosuppressive microenvironments (e.g., glioblastoma, ovarian cancer) and in earlier disease settings, such as neoadjuvant and adjuvant therapy, where immune modulation may be more effective. Additionally, next-generation inhibitors with improved pharmacokinetic properties and novel mechanisms of action-such as dual IDO1/TDO inhibitors or compounds targeting downstream kynurenine pathway effectors-are under preclinical and early clinical development. The integration of comprehensive immune monitoring and multi-omics profiling in ongoing trials may help identify predictive biomarkers and elucidate resistance mechanisms, potentially revitalizing the clinical application of IDO1 pathway modulation. While challenges remain, the continued exploration of IDO1 inhibition reflects its compelling biological rationale and the need for innovative approaches to overcome tumor immune evasion in cancer immunotherapy.

## 4 PROTACs: a paradigm shift in IDO1 targeting

**FIGURE 1:**
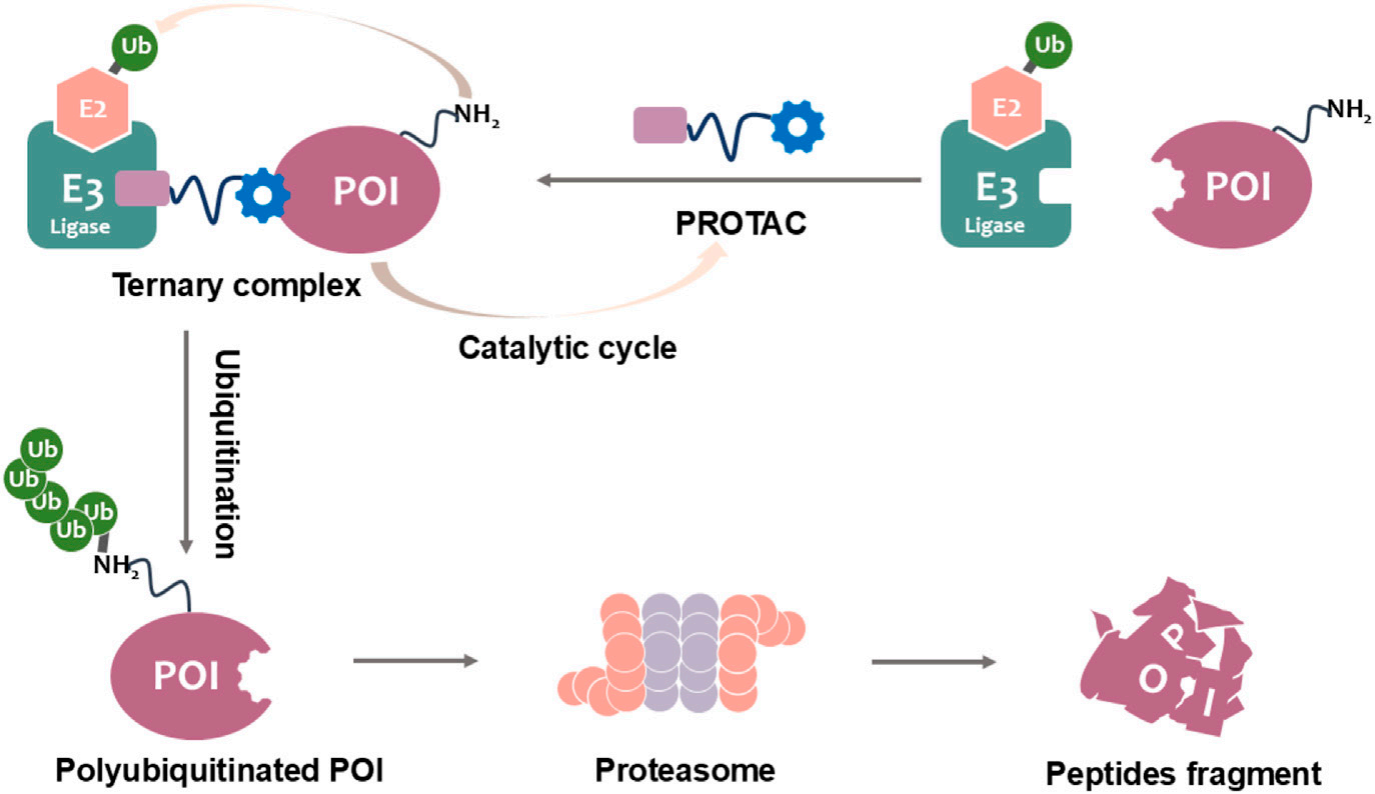
PROTAC-mediated degradation of target proteins through the UPS.

The development of PROTACs represents a groundbreaking advancement in IDO1-targeted cancer immunotherapy, offering a fundamentally distinct mechanism of action compared to conventional small-molecule inhibitors. These heterobifunctional molecules comprise three essential structural elements: (1) a high-affinity ligand that selectively binds IDO1, (2) a recognition domain for E3 ubiquitin ligase recruitment, and (3) a precisely engineered linker that optimizes spatial orientation between these components (Nalawansha and Crews, 2020; Yao et al., 2022). By exploiting the endogenous ubiquitin-proteasome system, PROTACs induce targeted polyubiquitination and subsequent degradation of IDO1 (Figure 1). This catalytic mechanism provides significant pharmacological advantages, including sustained target elimination at substoichiometric doses and reduced off-target effects relative to traditional occupancy-driven inhibitors. Importantly, PROTACs overcome key limitations of conventional approaches by effectively targeting both enzymatic and scaffolding functions of IDO1, while simultaneously addressing resistance mechanisms mediated by protein overexpression or mutations (Wang et al., 2023).

The selection of E3 ligase systems represents a critical determinant of PROTAC efficacy and tissue distribution. Current research has identified CRBN (cereblon) and VHL (von Hippel-Lindau) as particularly promising candidates, each offering distinct therapeutic advantages. CRBN-based degraders, frequently incorporating thalidomide analogs, demonstrate superior blood-brain barrier penetration, making them especially valuable for treating CNS malignancies (Wang et al., 2024). In contrast, VHL-based systems may provide enhanced specificity for peripheral tumors (Wan et al., 2020). Recent structural optimizations have yielded PROTACs with picomolar degradation potency and improved pharmacokinetic profiles, while emerging delivery platforms such as nanoparticle formulations address previous challenges in bioavailability. These technological advancements position PROTACs as versatile tools for comprehensive IDO1 inhibition, capable of disrupting both metabolic and non-metabolic immunosuppressive pathways in the tumor microenvironment.

## 5 Cutting-edge developments in IDO1 PROTAC technology

A landmark achievement in this field was reported in 2020 by Xie et al., who pioneered the first IDO1-directed PROTAC (compound 2c, Table 2) (Hu et al., 2020). This innovative molecule was strategically designed by conjugating the established IDO1 inhibitor epacadostat with a cereblon (CRBN)-binding ligand through a carefully optimized polyethylene glycol-based linker. The design process was informed by detailed structural biology studies that identified the solvent-exposed sulfamide group of epacadostat as an ideal attachment point for linker incorporation. This first-generation degrader demonstrated impressive biological activity, achieving near-complete (93%) target degradation at micromolar concentrations (DC_50_ = 2.8 μM) while maintaining moderate enzymatic inhibition (IC_50_ = 1.1 μM). Perhaps most significantly, it enhanced the antitumor efficacy of engineered T cells while exhibiting favorable selective cytotoxicity (IC_50_ = 37.4 μM against tumor cells), thereby validating the therapeutic potential of IDO1 degradation in cancer immunotherapy.

**TABLE 2:** Representative PROTACs targeting IDO1.

PROTAC	Target	Structure	Biological activity
compound 2c	IDO1	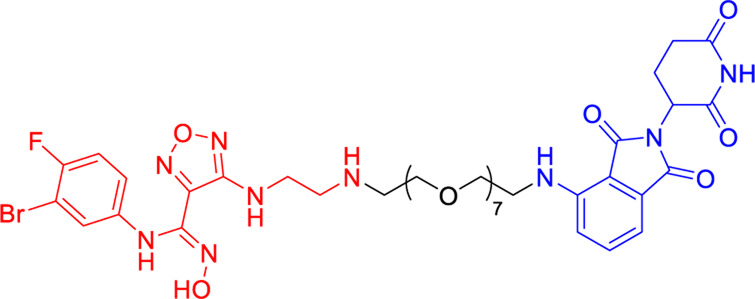	DC_50_ = 2.8 μM
SPNpro	IDO1	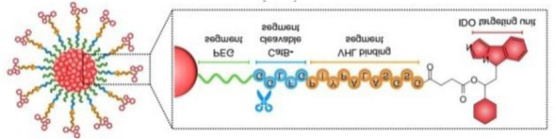	Effective
NU223612	IDO1	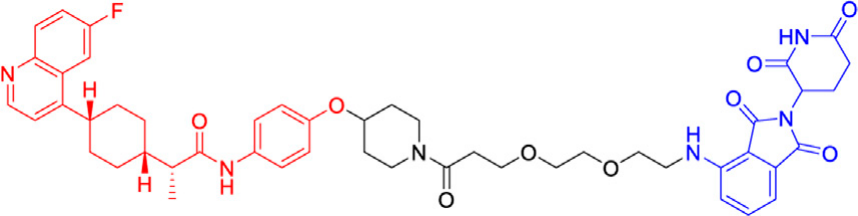	DC_50_ = 0.1 μM
NU227326	IDO1	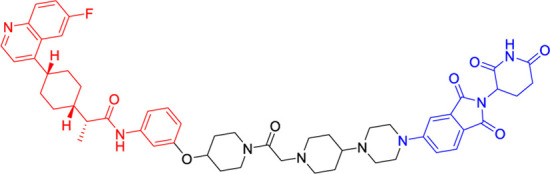	DC_50_ = 5 nM

Building upon this foundational work, subsequent research has dramatically expanded the capabilities of IDO1-targeting PROTACs through innovative molecular engineering approaches. A particularly notable advancement came in 2021 with the development of SPNpro (Table 2) by Pu and colleagues - a multifunctional semiconducting polymer nano-PROTAC that represents a paradigm shift in targeted protein degradation technology (Zhang et al., 2021). This sophisticated platform combines photodynamic therapy with tumor microenvironment-responsive PROTAC activation, creating a dual-action therapeutic system with several unique advantages. Under near-infrared light irradiation, SPNpro generates cytotoxic singlet oxygen that directly eliminates tumor cells while simultaneously inducing immunogenic cell death. The PROTAC component is selectively activated in the tumor microenvironment through cathepsin B-mediated cleavage, ensuring precise spatial and temporal control of IDO1 degradation. This innovative approach addresses several key challenges in cancer therapy, including off-target effects and limited tumor specificity. The sustained IDO1 degradation achieved by SPNpro effectively blocked the immunosuppressive kynurenine pathway *in vivo*, resulting in robust T cell activation and enhanced antitumor immunity. The combination of phototherapy-induced immunogenic cell death with PROTAC-mediated metabolic reprogramming created a powerful synergistic effect, demonstrating significant inhibition of both primary tumor growth and metastatic spread across multiple preclinical models.

The continuous refinement of IDO1-targeting PROTACs has led to the development of increasingly potent and therapeutically promising compounds with enhanced central nervous system penetration. Building upon earlier prototypes, Wang and Zhou, (2021) conducted comprehensive structure-activity relationship studies to optimize PROTAC design specifically for glioblastoma treatment, addressing the unique challenges of the blood-brain barrier and immunosuppressive brain tumor microenvironment (Bollu et al., 2022). Their lead compound, NU223612 (Table 2), demonstrated breakthrough efficacy in orthotopic glioblastoma models, achieving robust IDO1 degradation within the intracranial tumor niche - a feat particularly notable given the historically poor penetration of most therapeutic agents into the central nervous system. Detailed mechanistic characterization revealed this PROTAC’s multifaceted action, showing complete ablation of both IDO1’s canonical enzymatic function and its less-characterized non-enzymatic roles in NF-κB pathway activation and other pro-tumorigenic signaling cascades. These comprehensive pharmacological effects translated to significant survival benefits in preclinical glioblastoma models, marking a major advancement in brain tumor immunotherapy.

The most recent breakthrough in this series, reported in 2025, represents the culmination of iterative PROTAC optimization efforts (Monsen et al., 2025). The next-generation compound NU227326 (Table 2) exhibits unprecedented picomolar-range degradation potency (DC_50_ = 5 nM), representing a >500-fold improvement over first-generation IDO1 degraders. This exceptional potency is complemented by remarkably sustained target suppression, maintaining >90% IDO1 degradation for over 48 h in human glioblastoma cell lines - a critical feature for overcoming the rapid protein turnover often observed in aggressive tumors. The compound’s pharmacokinetic profile has been carefully engineered to balance blood-brain barrier penetration with systemic stability, addressing one of the most persistent challenges in neuro-oncology drug development. Furthermore, NU227326 demonstrates exquisite selectivity for IDO1 over related enzymes like TDO, minimizing off-target effects while comprehensively disrupting the kynurenine pathway. These properties collectively position NU227326 as a potential best-in-class therapeutic candidate, not only for glioblastoma but potentially for other IDO1-dependent malignancies where sustained, complete pathway inhibition is required for optimal therapeutic effect.

These successive advancements highlight the remarkable evolution of PROTAC technology from initial proof-of-concept molecules to highly optimized therapeutic candidates. The field continues to progress rapidly, with ongoing research focusing on several key areas: 1) refinement of structure-degradation relationships to enhance potency and selectivity, 2) development of innovative delivery strategies to improve tissue penetration and pharmacokinetic properties, and 3) exploration of combination therapies to maximize therapeutic efficacy. The application of PROTAC technology to IDO1 inhibition has not only provided new tools for cancer immunotherapy but has also established a framework for targeting other immunometabolic pathways in oncology. As our understanding of protein degradation mechanisms deepens and molecular engineering capabilities advance, PROTAC-based therapies are poised to make significant contributions to the next-generation of cancer treatments.

## 6 Conclusion

The exploration of IDO1 as a therapeutic target in cancer immunotherapy has evolved from early small-molecule inhibitors to advanced protein degradation strategies. Despite promising preclinical results, first-generation IDO1 inhibitors exhibited limited clinical efficacy, as highlighted by the disappointing outcomes of the ECHO-301/KEYNOTE-252 trial. This underscores the complexity of tryptophan metabolism in immune regulation, where compensatory pathways (e.g., TDO) and non-enzymatic functions of IDO1 contribute to therapeutic resistance. To overcome these challenges, next-generation approaches such as PROTACs have emerged, enabling complete IDO1 degradation and eliminating both its catalytic and scaffolding roles. Innovations like SPNpro nano-PROTACs further enhance tumor specificity and combinatorial potential, demonstrating efficacy even in resistant malignancies like glioblastoma.

Future trials must prioritize robust biomarker strategies to optimize patient selection. IDO1 expression levels, measured via immunohistochemistry or RNA sequencing, could identify tumors reliant on this pathway. Additionally, the kynurenine/tryptophan (Kyn/Trp) ratio in serum or tumor tissue may serve as a dynamic pharmacodynamic marker, reflecting IDO1 activity and predicting therapeutic response. Integrating multi-omics analyses (e.g., transcriptomic or metabolomic profiling) could further refine patient stratification by uncovering compensatory mechanisms, such as TDO upregulation or immune checkpoint co-expression (e.g., PD-L1, CTLA-4). Such precision approaches may resurrect the clinical potential of IDO1-targeted therapies by enriching for responsive populations.

Given the immunosuppressive interplay between IDO1 and other pathways, combination therapies hold significant promise. Co-targeting PD-1/PD-L1 may synergize with IDO1 degradation, as both pathways converge on T-cell exhaustion. Preclinical evidence suggests that PROTAC-mediated IDO1 removal enhances checkpoint inhibitor efficacy by reshaping the tumor immune microenvironment. Chemotherapy combinations could also exploit immunogenic cell death to amplify antitumor immunity. Furthermore, dual metabolic modulation-such as pairing IDO1 degraders with adenosine pathway inhibitors or glutaminase blockers-may address tumor plasticity. Systematic evaluation of these combinations, guided by mechanistic biomarkers, will be critical for clinical translation.

Key challenges include optimizing PROTAC delivery, particularly to immune-privileged sites like the CNS, and minimizing off-target effects. Advances in nanoparticle formulations or conditional activation systems (e.g., tumor microenvironment-responsive PROTACs) may improve therapeutic indices. Additionally, the IDO1 experience offers a blueprint for targeting other immunometabolic nodes (e.g., ARG1, CD73) via degradation. As the field progresses, integrating deep molecular profiling with innovative drug modalities will be essential to unlock the full potential of cancer immunotherapy.
